# Bone Regeneration Using MMP-Cleavable Peptides-Based Hydrogels

**DOI:** 10.3390/gels7040199

**Published:** 2021-11-05

**Authors:** Weikai Chen, Ziyang Zhou, Dagui Chen, Yinghua Li, Qin Zhang, Jiacan Su

**Affiliations:** 1 Institute of Translational Medicine, Shanghai University, Shanghai 200444, China; chenweikai0819@shu.edu.cn (W.C.); Zzy21724887@shu.edu.cn (Z.Z.); dagui1106@shu.edu.cn (D.C.); 2 School of Medicine, Shanghai University, Shanghai 200444, China; 3 School of Environmental and Chemical Engineering, Shanghai University, Shanghai 200444, China; 4 School of Life Sciences, Shanghai University, Shanghai 200444, China; 5 Department of Trauma Orthopedics, Changhai Hospital, Naval Medical University, Shanghai 200433, China; 6 Shanghai Clinical Research Center for Aging and Medicine, Shanghai 200040, China

**Keywords:** MMP-cleavable peptides, crosslinking, hydrogels, degradation, bone regeneration

## Abstract

Accumulating evidence has suggested the significant potential of chemically modified hydrogels in bone regeneration. Despite the progress of bioactive hydrogels with different materials, structures and loading cargoes, the desires from clinical applications have not been fully validated. Multiple biological behaviors are orchestrated precisely during the bone regeneration process, including bone marrow mesenchymal stem cells (BMSCs) recruitment, osteogenic differentiation, matrix calcification and well-organized remodeling. Since matrix metalloproteinases play critical roles in such bone metabolism processes as BMSC commitment, osteoblast survival, osteoclast activation matrix calcification and microstructure remodeling, matrix metalloproteinase (MMP) cleavable peptides-based hydrogels could respond to various MMP levels and, thus, accelerate bone regeneration. In this review, we focused on the MMP-cleavable peptides, polymers, functional modification and crosslinked reactions. Applications, perspectives and limitations of MMP-cleavable peptides-based hydrogels for bone regeneration were then discussed.

## 1. Introduction

Bone tissue regeneration in orthopedic and maxillofacial surgery remains a common challenge [1]. Trauma, tumors, infectious diseases, biochemical disorders, congenital disorders or abnormal skeletal development are the cause of bone defects, resulting in functional, esthetic and psychological defects in patients [2]. Natural healing of skeletal structure is relatively limited and requires assistance during pathological conditions such as severe injuries, osteoporosis, osteosarcoma and infection [3]. Autogenous bone was identified as the gold standard for bone defects and retained perfect biocompatibility, but it could not fully satisfy the requirements due to low yield, iatrogenic injury and risk [4]. Other solutions such as allografts, xenografts and bone substitute materials hold corresponding shortcomings in terms of, for example, immune response, infectious risk and disease transmission [5,6]. Therefore, a further sustainable and high-yielding strategy is required, which leads us to tissue engineering methods. Numerous studies have recently introduced bioactive scaffolds and their interaction with adjacent bony tissues, and hydrogels have received attention due to their excellent biocompatibility, biodegradability and plasticity [7,8,9].

With their hydrophilic polymeric networks, hydrogels are considered the most promising polymer scaffold in bone tissue engineering [10], and the modification of their permeability and stiffness enables substance exchanges and cell function [11,12]. As the basis and guiding principle of bone regeneration, the degradation behavior of hydrogels is directly related to the speed and quality of bone repair [13]. Specifically, hydrogels in bone regeneration should be constructed by biocompatible materials and hold enough stability for cell activity at an early stage [14,15]. Along with cell growth and microstructural remodeling, biodegradation of hydrogels is required to create appropriate space for the incoming inhabitants. Despite the natural and synthetic polymers used in their preparation, the degradation solution of hydrogels mainly takes into account temperature, pH, light irradiation, ultrasound and enzymes, among other aspects. [16,17,18]. Among them, enzymatically responsive hydrogels are well-recognized at present for their controlled and tunable degradation adapted to in vivo circumstances [19,20].

Response and adaption under environmental variation are intrinsic properties of all biosystems, as well as biomaterials [21]. The transformation of spatial configurations, physical properties or structural stability under proper stimulation helps in the degradation of bone fillers and the release of bioactive cargoes. Enzymes were valued as a promising trigger for novel responsive polymers, considering their biological origin, efficiency and high selectivity [22]. Leading-edge research reported that clustered regularly interspaced short palindromic repeats (CRISPR)-associated enzymes could be utilized to cleave DNA cargoes in responsive hydrogels and for the delivery of genetic information [23]. Remarkably, enzyme levels vary with in vivo microenvironments and biological behaviors, and this variation was used in a novel strategy that integrates enzymatic reaction and controlled release [24]. For instance, a smart hydrogel constructed by glutathione-modified collagen and MMP-cleavable peptide targeted myocardial infarction and ameliorating myocardium remodeling in vivo in a “release on-demand” manner [25]. Particularly, it was revealed that MMPs are involved in bone remolding. Thus, the MMP-cleavable peptides-based hydrogels are promising candidates for bone tissue engineering.

The growing demand for MMP-cleavable peptides-based hydrogel as a platform for biomedical applications exhibits a strong need for a timely review on a wide range of their fabrication and applications in bone repair. This review discusses the latest advances in MMP-cleavable peptides-based hydrogels for biomedical applications in bone regeneration. The MMP-cleavable peptides are introduced as crosslinkers for hydrogels. The three commonly used MMP-cleavable peptides-based hydrogels, including Poly(ethylene glycol) (PEG)-, hyaluronic acid (HA)- and chitosan (CS)-based hydrogels, are then highlighted. The advantages and limitations of using these hydrogels along with their different synthesis methods are summarized. Additionally, their most recent advances in the field of bone science, including hydrogel-based 3D in vitro models and bone healing, are subsequently reviewed. Finally, the current challenges and future perspectives of MMP-cleavable peptides-based hydrogels are briefly discussed.

## 2. MMP-Cleavable Peptides

### 2.1. Definition of MMP-Cleavable Peptides

Due to the bioactivates and biological function of MMPs, they can be used as triggers in degradable biomaterials. MMP-cleavable peptides, which are composed of several amino acids in a specific sequence and are sensitive to different MMPs, were previously synthesized and incorporated into functional hydrogels [26]. The peptides mimic the natural ECM and could be recognized and degraded by MMPs in the cleavage site. For example, the most commonly used sequence (GPQG↓IWGQ), where ↓ suggests the cleavage site, is sensitive to MMP-2, MMP-9 and MMP-14 [27]. The flanking linker sequence has been regarded as a popular solution for peptide modification. For example, the GCRD sequence was utilized to synthesize the GCRD-VPMS↓MRGG-DRCG complex. In this regard, the water-solubility of the peptide was upregulated via the hydrophilic arginine (R), and the thiol group-based crosslinker was introduced due to the existence of cysteine (C), glycine (G) and aspartic acid (D) as spacers [17].

MMP-cleavable peptides-based hydrogels have been designed and applied in tissue engineering for decades. MMP-2 and MMP-9 could be manufactured by MSCs and endothelial cells to degrade ECM during bone resorption and formation [28,29]. Hence, hydrogels crosslinked with the MMP-cleavable peptide (GKKC-GPQGIWGQ-CKKG) have been commonly designed and implanted into bone defect sites of mice to promote bone regeneration [30]. In addition, MMP-7 is commonly over-expressed in the tumor microenvironment, and is identified as promising stimuli. The MMP-7-sensitive peptide sequence (CGG-PLGLA-GGC) containing thiol groups was applied to crosslink maleimide (MAL) groups in polymers inside specific hydrogels [31]. A short peptide-based, self-assembled Ac-I3SLKG-NH2 hydrogel was introduced by Chen et al., which is sensitive to MMP-2 and could be degraded into Ac-I3S and LKG-NH2. The anticancer peptide-G3 entrapped into the hydrogel could be released in a “cell-demand” manner, and thus, inhibit the tumor growth along with the hydrogel degradation that overexpressed MMP-2 by HeLa cells are exposed to [32]. Thus, MMP-cleavable peptides have exhibited exciting potential for biomedical tissue engineering.

### 2.2. Kinetic Parameters of MMP-Cleavable Peptides

**Figure 1:**
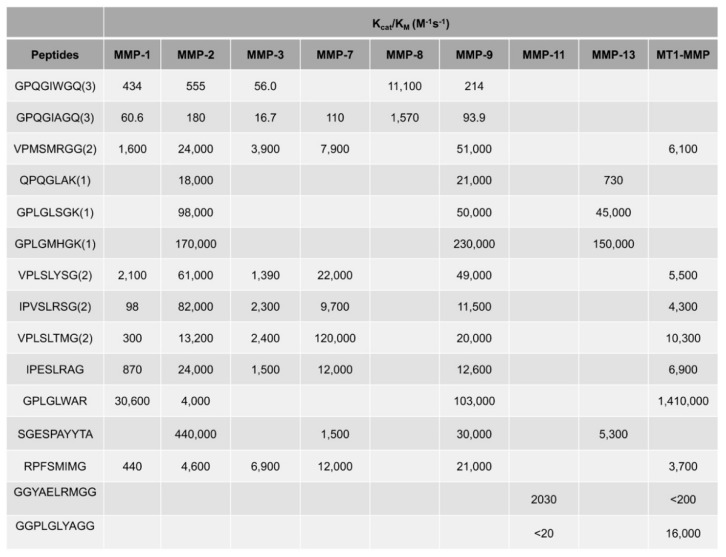
Kinetic parameters for MMP-cleavable peptides [33,38]. Reprinted with permission from Copyright © 2010 Elsevier Ltd, and Copyright © 2016 Elsevier Ltd.

In order to quantize the degradation kinetics of MMP-cleavable peptides, Michaelis–Menten analysis was adapted to measure the kinetic parameters of the substrates by a fluorometric experiment [33]. The two major kinetics parameters K_M_ and k_cat_ were calculated by fitting rate and substrate concentration according to the Michaelis–Menten equation (Figure 1). K_M_ is the Michaelis constant, which is related to enzymes, and k_cat_ stands for the ability of enzymes to catalyze substrates. For example, MMPs were cocultured with different substrates at 30 °C in buffer solution. Then, the degradation rates were monitored by measuring the fluorescence intensity [34]. 

It is well known that the degradation rates depend on several factors, such as peptide substrate sequences, and the type and concentration of MMP. Compared to GPQGIWGQ, the kcat value of GPQGIAGQ is increased, meaning that the degradable rate has been accelerated by transferring an amino acid substitution (A to W) [33]. In addition, different peptides are optimized for different MMPs. For example, the peptide (VPMSMRGG) is optimal for MMP-1 degradation and shows a faster degradation rate than GPQGIAGQ or GPQGIWGQ [35]. Furthermore, the degradable rates of sequence varied for different types of MMPs [36,37,38]. As a result, the degradation duration of different sequences could vary from less than 2 days to more than 10 days.

## 3. General Materials for MMP-Cleavable Peptides-Based Hydrogels

### 3.1. Hyaluronic Acid

**Figure 2:**
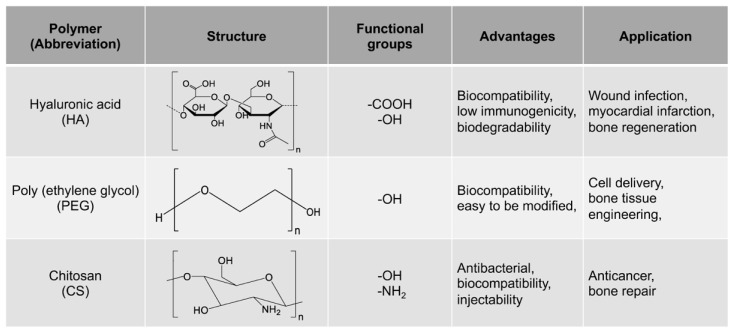
Polymers for MMP-cleavable peptides-based hydrogels.

Hyaluronic acid (HA) is a kind of non-sulfated glycosaminoglycan, which is found from the vitreous body of the eye to the extracellular matrix (ECM) of cartilage tissue, throughout the body. Because of its high biocompatibility, low immunogenicity, biodegradability and ability to interact with extracellular information molecules [39,40,41], HA is widely used in medical products, including engineering hydrogels [42,43], cell therapy and three-dimensional (3D) cell culture [44] (Figure 2). For example, Zhu et al. prepared antibacterial sanguinarine/gelatin microsphere/dextran-HA hydrogels by oxidizing glucan and amino HA [45]. Its application in the treatment of full-thickness burn infections in the standard deviation rat model was evaluated. It was found that the hydrogel had a longer drug release time, as well as effective antibacterial activity and wound regeneration ability. It can effectively inhibit the formation of scars after burn infection.

In addition, hyaluronic acid (HA) has bifunctional modification sites and multiple active groups, which can be easily chemically modified to meet the functional needs of different situations [46]. Wang et al. modified HA with hydrazides or aldehydes and mixed them to form shear-thinning and self-healing hydrogels through dynamic hydrazone bonds [47]. Then, the HA was further modified with β-cyclodextrin to encapsulate cholesterol-modified siRNA and limit the passive diffusion of siRNA, and injectable and protease-degradable hydrogels were prepared. According to the proteolytic activity after myocardial infarction, the hydrogel can release siRNA as needed, silence the expression of MMP2, and then affect the function of cardiac cells, resulting in the improvement of hemodynamic function.

Besides, HA hydrogel is also widely used in the field of bone tissue regeneration [46]. Ren et al. designed and synthesized a biomimetic hydrogel system based on Maleimide-modified HA [48]. With the MMP-sensitive peptide (GCRDGPQGI↓WGQDRCG) being used as the cross-linker, the hydrogel was prepared by coupling the collagen mimetic peptide (GPO)8-CG-RGDS with HA. It was found that the hydrogel could mimic the properties of collagen and was sensitive to MMP-2. In addition, it could also increase the expression of collagen alpha 1 (II), aggrecan and SOX9 genes in bone marrow mesenchymal stem cells, which may have the potential to induce BMSCs to differentiate into cartilage and inhibit the hypertrophic phenotype during differentiation.

It was reported that HA-based hydrogels that are sensitive to MMPs but not sensitive to hydrolysis can be prepared by crosslinking Maleimide-modified HA macromolecules with MMP-cleavable peptides [49]. Feng et al. designed and synthesized two kinds of hydrolysis-insensitive HA hydrogels, compared the effects of MMP-sensitive hydrogels and MMP-insensitive hydrogels on human mesenchymal stem cells (hMSCs), and eliminated the confounding factors of hydrogel degradation due to hydrolysis [50]. Studies have shown that the cell-mediated degradation of matrix metalloproteinases in hyaluronic acid hydrogel promotes the cartilage formation of hMSCs but inhibits the hypertrophy of hMSCs.

### 3.2. Poly (Ethylene Glycol) (PEG)

Poly (ethylene glycol) (PEG) is a hydrophilic polymer that has the characteristics of biocompatibility and bioinertia, and it can support cell growth after the addition of the appropriate protease-sensitive connectors and cell adhesion sites [51]. Therefore, PEG hydrogel is a promising synthetic hydrogel. PEG hydrogels have interconnected microporous networks that provide continuous nutrient flow, cell growth and vascularization of engineering tissue (Figure 2). Studies showed that PEG hydrogel helps to maintain the phenotype of natural heart valve cells [52], optimize cell viability and morphology [53], and promote the production of extracellular matrix [54]. Dai et al. prepared a kind of stromal cell-derived factor-1-α-loaded MMP degradable PEG hydrogel [55]. The experimental data show that the hydrogel has good biocompatibility, can promote the recruitment of mesenchymal stem cells, can promote the phenotypic polarization of M2 macrophages, and has good tissue remodeling ability. The hydrogel can also improve the adhesion, activity and proliferation of bone marrow mesenchymal stem cells (BMSCs) and promote the differentiation of BMSCs into valvular interstitial-like cells.

In addition, PEG hydrogels can be modified to meet the needs of specific applications in vitro and in vivo [8,56,57,58,59]. Metzger et al. cross-linked Streptavidin with PEG to prepare hydrogel, which can release immobilized growth factor (GF) and does not depend on the degradation of hydrogel [60]. Research data show that through the appropriate design of the release system, GF can be released by PEG hydrogels in a soluble form that is more effective than the supplementary cell culture medium for local delivery.

Moreover, PEG hydrogel is widely used in cell delivery and bone tissue engineering [61]. Sridhar et al. developed a peptide- and protein-functionalized PEG hydrogel. After being co-cultured with the hydrogel for 14 days, chondrocytes significantly increased the deposition of glycosaminoglycans and collagen, maintained a high level of activity, and produced a more widely distributed matrix. This shows that hydrogel can promote the production of cartilage matrix [62].

It was reported that PEG hydrogel can be used as a blank skeleton, in which multiple scaffolds with various functions can be systematically introduced into the scaffold to allow integrin binding [63], proteolysis and degradation [33,51], and even local isolation of growth factors [64]. Therefore, PEG hydrogels with specific material compositions can be used to guide mesenchymal stem cells to differentiate into specific types of chondrocytes [65]. Nguyen et al. designed and synthesized a three-layer composite hydrogel, based on PEG, that was doped with chondroitin sulfate, metalloproteinase-sensitive peptides and HA [66]. The results show that the hydrogel can not only induce MSCs to differentiate into chondrocytes, but also customize the phenotype and matrix production pattern of differentiated cells according to the specific region of articular cartilage by changing the material composition.

### 3.3. Other Polymers

Chitosan-based hydrogels were chosen as an embolic matrix because of their good biocompatibility, biodegradability, injectability and adhesion at room temperature [67,68] (Figure 2). They can also rapidly undergo sol–gel transition at body temperature. Zehtabi et al. designed and synthesized Chitosan-Doxycycline hydrogel [69]. The hydrogel can be injected through a microcatheter and has gelation and mechanical properties that are rapid enough to block the tubular structure under physiological pressure. The hydrogel can also release bioactive Doxycycline (DOX), inhibit the MMP-2 activity of human glioblastoma cells, remove endothelium and induce vascular thrombosis. Preliminary in vivo tests on porcine renal arteries showed that the success rates of immediate and delayed embolization were 96 and 86%, respectively. Gustafson et al. prepared a hydrogel that can be degraded by matrix metalloproteinases by modifying the skeleton of silk-elastin-like protein polymers (SELPs) with matrix metalloproteinase-sensitive peptides [70]. The results showed that MMP-2 and MMP-9 increased protein loss by 63 and 44% respectively, increased the release of 65 and 95% MMP-sensitive hydrogels, and decreased the compression modulus by 41 and 29%, respectively. It was suggested that the SELP reacted by matrix metalloproteinases may transport bioactive substances locally where MMPs are overexpressed. Fonseca et al. modified sodium alginate with matrix metalloproteinase-sensitive peptides to prepare an injectable hMSC-MMP-sensitive alginate saline hydrogel [71]. The experimental results show that the hydrogel can be used as a local repository of cells to promote tissue regeneration and provide protection for transplanted cells at the same time.

Moreover, the hydrogels prepared by some synthetic polymers also have excellent properties. For example, Qian et al. synthesized poly (propylene sulfide) 120 (PPS120), which has hydrophobicity- and reactive oxygen species (ROS)-quenching and H_2_O_2_-responsive abilities [72]. Reactive oxygen species depletion hydrogels were prepared by embedding PPS120 with Matrix metalloproteinase (MMP)-responsive triglycerol monostearate. The hydrogel can release Cur in cerebrospinal fluid, effectively reduce the ROS level of astrocytes in vitro and in the human brain, and effectively protect the blood–brain barrier and improve brain edema. In the work of Chung et al, based on the environmentally friendly poly (N-isopropylacrylamide-co-acrylic acid) hydrogel, the degradable cross-linking agent of matrix metalloproteinase-13 (MMP-13) and the peptide-containing integrin-binding domain (Arg-Gly-Asp) were combined [73]. The experimental results show that the hydrogel can significantly promote bone regeneration in a rat femoral ablation model.

## 4. Synthesis of MMP-Cleavable Peptides-Based Hydrogels

### 4.1. Polymer Modification

The thiol groups of cysteine usually act as a crosslinker in MMP-cleavable peptides. Although some MMP-cleavable peptides could be crosslinked with polymers by introducing chemical groups via the grafting of amino acids to peptides, tt is easier to introduce some functional groups into the polymers to construct hydrogels with the amino acid sequences. Several common methods of polymer modification are discussed below.

#### 4.1.1. Maleimide Functionalization

Maleimide (MAL) groups are famous chemical fragments and are widely used as small molecule linkers in medical chemistry and biochemistry [74]. Their application fields vary from multifunctional polymers to biomaterials due to their fast kinetics, which means the polymers crosslink quickly and form hydrogels in situ [75,76], a light-mediated reagent that may be toxic is not necessary for the reaction system [77], and the competing side-reactions are minimized by the high specificity and reaction efficacy [78]. There are several approaches for MAL group introduction. The carboxyl group of hyaluronic acid (HA) was activated after adding N-(3-Dimethylaminopropyl)-N-ethylcarbodiimide hydrochloride (EDC·HCl) and N-hydroxysulfosuccinimide (Sulfo-NHS). Then, the MAL group of N-(2-Aminoethyl) maleimide trifluoroacetate salt (AEM) could be grafted to HA via the amino-carboxyl reactions [48]. Therefore, chondroitin sulfate, which also contains a carboxyl group, could employ the MAL group in the same way.

#### 4.1.2. Norbornene Functionalization

Norbornene (NB) groups, which are also molecule linkers, have attracted increasing attention because their photo-crosslink property and have been widely introduced into biomaterials for use as a bioink in bioprinting [79,80,81]. It is well-known that the photochemical reaction of the NB group holds a speedy reaction rate under physiological pH and temperature, and that the reactions could occur at relatively low radical concentrations [82]. These advantages demonstrate that introducing the NB group into biomaterials might be a promising solution in biomedicine and tissue engineering. Gelatin is a natural polymer, which exhibits cell-interactive properties, and could be easily modified due to its diverse chemical groups, including -OH, -COOH and -NH2. Therefore, gelatin could employ an NB group using 5-norbornene-2-carboxylic acid in the reaction of the carboxylic acid and the primary amines [82]. The norbornene derivant could also be utilized in NB group insertion; Guo et al. synthesized norbornene-collagen that was obtained from acidic collagen after reacting with carbic anhydride [83]. 

In addition, PEG, which is identified as one of the most common synthesis polymers, also combines with the NB group under the appropriate circumstances. Eight-arm PEG-hydroxyl, dissolved in dichloromethane (DCM) with pyridine and 4-Dimethylaminopyridine (DMAP), could introduce NB groups via an overnight reaction with 5-norbornene-2-carboxylic acid and N,N’-dicyclohexylcarbodiimide under nitrogen conditions [84]. The hydrogel could be formed with MMP-cleavable peptides under ultraviolet light (UV) with lithium phenyl-2,4,6-trimethylbenzoylphosphinate (LAP) and elevated alkaline phosphatase (ALP) activity. As a result, it could be developed as a prospective biomaterial for bone regeneration.

#### 4.1.3. Vinyl-Sulfone Functionalization

Vinyl-sulfone (VS) groups are widely used in hydrogels as a non-zero-length cross-linker and offer such advantages as physiological and biocompatible reaction conditions, non-initiator gelation, high mechanical stability, and reasonable specificity [85,86]. Therefore, VS groups are considered as an optimal choice to construct injectable hydrogels [9]. On the other hand, when comparing with MAL groups, VS groups exhibit a much slower reaction rate, which provides abundant time for the mixture of reactive precursors. Furthermore, VS can react with amine or thiol groups of peptides [87,88]. Nowadays, VS groups have been introduced to more and more polymers, such as PEG, HA, dextran, gellan gum and so on [89,90]. It is well known that VS could be deprotonated in strongly alkaline condition. Thus, Dextran could employ a VS group in NaOH solution after adding divinyl sulfone via the Michael addition reaction with -OH and C=C [87]. This reaction could be stopped by lowering the pH. Different concentrations of RGD peptides were grafted to Dextran-VS via a thiol-vinyl sulfone reaction. The Dextran-VS-based hydrogel demonstrated that a low concentration of RGD (0.1%) was enough for cell adhesion. The polymers carrying -OH groups could be introduced to the VS groups in the same way [91].

#### 4.1.4. Other Functionalization

In addition to what has been mentioned above, there are varieties of ways to modify the polymers. The fact that stem cell technology has been used widely for tissue regeneration and biomaterial design represents a significant development. As the reaction of gelatin is important for cell encapsulation in the hydrogel, Paez et al. utilized the methylsulfonyl (MS) and thiols groups to form a hydrogel with a suitable reaction kinetic for cell encapsulation [92]. MS groups could be introduced into PEG via the reaction of PEG-NHS and an intermediate, which were obtained from MS-coupled Boc-glycine. The hydrogel exhibited hydrolytic stability and biocompatibility, but it could be easily degraded by MMPs due to the MMP-cleavable peptides crosslinked with PEG-MS via thiol-MS reaction. 

In addition, acrylate groups are also employed to modify the polymers. Acrylate functionalized hyaluronic acid (HA-AC) hydrogel was developed to deliver genetic information for local regulation [93]. Acrylate groups were introduced into HA via the Michael addition reaction after the carboxyl groups of HA reacted with adipic dihydrazide (ADH). HA-AC could be crosslinked with cysteine of MMP-degradable peptides to form a hydrogel via a Michael addition reaction in the presence of poly(ethylene imine) (PEI), which transfers DNA. The influence factors of transgene expression, such as matrix stiffness and RGD concentration, have also been investigated. The hydrogel was a promising way to deliver genes during in vivo gene therapy.

### 4.2. Chemical Reactions Using MMP-Cleavable Peptides

#### 4.2.1. Click Chemistry Reaction

**Figure 3:**
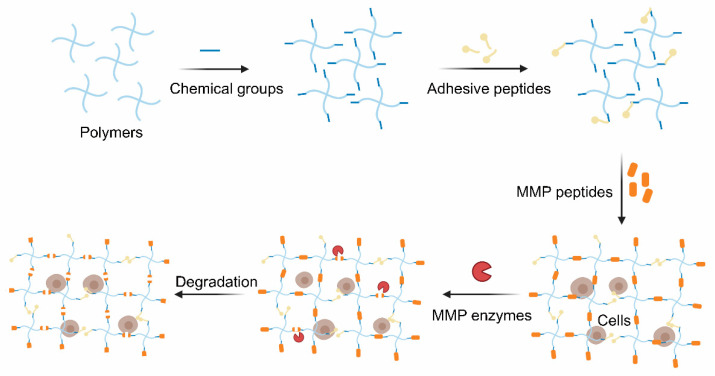
Preparation and degradation of MMP-cleavable peptides-based hydrogels.

The click chemistry reaction is inspired by nature and boasts mild reaction conditions, and also has high specificity, rich yielding and a speedy reaction rate [94,95]. In particular, it is biorthogonal and widely used in cell therapy with few side reactions [96]. Cysteine is commonly grafted into peptides since its thiol group and alkenes groups are rarely found in nature. Such peptides are extensively used to crosslink the polymers possessed alkene groups (typically the norbornene groups) to form hydrogels via the thiol-ene photo-click chemistry reaction between the thiol group and the alkene groups with cytocompatible light initiation. The reaction, which is mediated by light, starts with radical initiation upon irradiation to form a thiyl radical [97]. Furthermore, the hydrogels are polymerized in a step-growth manner. As a result, the hydrogels exhibit a spatiotemporally controlled gelation behavior and excellent cell encapsulation ability [98]. MMP-sensitive PEG-based hydrogels were identified, and they were found to be formed via the click reaction between 4-arm PEG-modified with norbornene groups and MMP-cleavable crosslinker (KCGPQG↓IWGQCK) [62]. Cells and growth factors were co-encapsulated into the hydrogel and functioned well based on the biocompatibility of this polymer (Figure 3). 

This reaction is usually applied in 3D printing due to its mild reaction condition and fast gel rate [99]. For example, collagen was found to possess many inherently useful properties for regenerative medicine, and it has also been widely used in the 3D printing field. Guo et al. developed a norbornene-functionalized collagen-based hydrogel, which acted as a bio-ink and exhibited cell viability, spreading and proliferation properties [83]. The printability property, which is critical for 3D printing, was tested using different printing methods. As a result, it was demonstrated that the norbornene-functionalized collagen bio-ink showed potential prospects in bioprinting [83].

Nevertheless, there are still some issues that should be precisely considered when adopting the thiol-ene photo-click reaction. In particular, the reaction may generate free-radical species under light exposure, and might be lethal to adjacent proteins. Additionally, as a result of the infeasibility of light exposure in specific tissues or organs, its application is partly limited in clinical contexts.

#### 4.2.2. Michael Addition Reaction

The Michael addition reaction is also biorthogonal and takes place in alkaline conditions. Maleimide, acrylate, methacrylate and vinyl sulfone groups are the common groups that react with peptides in Michael addition reactions [100]. The mechanism of the Michael addition reaction is that the thiolate anion coming from the deprotonated thiol reacts with maleimide and creates the intermediate. Then, the intermediate provides the object product after deprotonating an additional thiol [101]. It is suitable for cell encapsulation due to the mild reaction condition, fast kinetics, spontaneous initiation and 3D network [102]. An MMP-degradable hydrogel was synthesized by crosslinking MMP-cleavable peptides with PEG-MAL, which acted as the backbone of the polymer [103]. Biological properties of this hydrogel were tested after cell loading. As a result, this enzyme-degradable hydrogel is claimed as a promising biomaterial for stem cell delivery [104]. Similarly, 4-arm PEG-SH was crosslinked with MMP-sensitive peptide modified with MAL to construct novel MMP degradable hydrogels [105]. 

The Michael addition reaction is also utilized in drug delivery and the releasing of on-demand materials [106]. According to Guo et al., Diacrylate modified 8-arm PEG was crosslinked with MMP-sensitive peptide (CGPQG↓IWGQC) via the Michael addition reaction [107]. Cargoes could be released under the presence of MMPs, and it was found that the release kinetics may be adjusted with different drug loading methods and environmental MMP concentrations.

However, this type of reaction may lead to unexpected off-stoichiometric reactions of monomers [98]. Furthermore, the spontaneous initiation under basal conditions made it difficult to control the spatiotemporal process, which might limit its application in tissue engineering.

#### 4.2.3. Other Reactions

Different methods for connecting polymers and peptides have recently been introduced. Following guest–host chemistry methods, Rodell et al reported a noncovalent injectable hydrogel; this was self-assembly crosslinked via the guest–host complexation of adamantane (guest, Ad) and β-cyclodextrin (host, CD) [108]. Ad was coupled to MMP-degradable peptides (VPMS↓MRGG) and CD was bound to HA, respectively. The hydrogel exhibited shear-thinning characteristics, selective proteolytic degradability and prolonged target retention.

**Figure 4:**
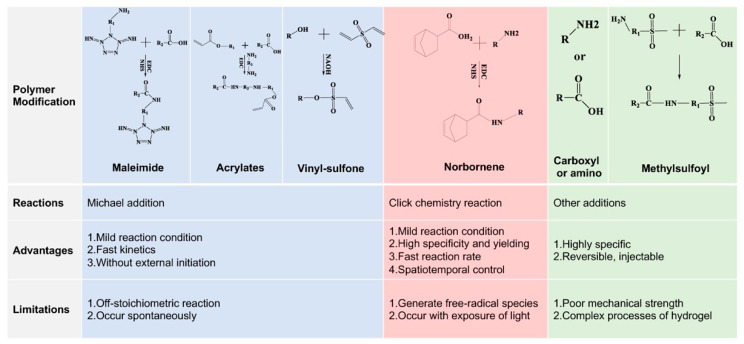
Polymer modifications, reactions, advantages and limitations of MMP-cleavable peptides-based hydrogels.

Hydrogels consisting of glutamine-peptide-functionalized 8-arm PEG-VS and MMP-Lys-peptide-modified chondroitin-sulfate-MAL could be crosslinked by transglutaminase factor XIII, under physiological conditions, without any other initiators [109]. The hydrogel exhibited a highly specific crosslink mechanism that could be used as a modular method to form hydrogel for regenerative medicine. Above all, there are still many other reactions to form MMP-cleavable hydrogels; those that are described in this paper are a selection of the popular strategies used in hydrogel preparation (Figure 4).

## 5. Applications of MMP-Cleavable Peptides-Based Hydrogels in Bone Science

### 5.1. Hydrogel-Based 3D In Vitro Models for Studying Cellular Responses

#### 5.1.1. Enhancing Osteogenic Capacity

**Figure 5:**
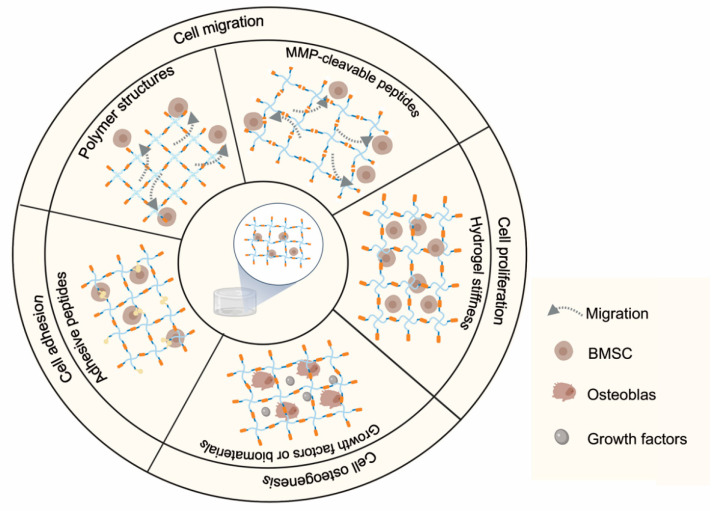
Illustration diagram of MMP-cleavable hydrogel-based 3D in vitro models for studying cellular responses.

Hydrogels, which possess a three-dimensional network and a high amount of water, are prospective biomaterials for cell encapsulation. Cell behaviors in hydrogels have been widely investigated in recent years, including cell adhesion, spreading, proliferation and differentiation (Figure 5) [110]. Many efforts have been made to further increase osteogenesis. The most common approach is to incorporate biomaterials or biomolecules into the hydrogel. Growth factors have been loaded in hydrogels to promote osteogenesis. Bone morphogenetic protein (BMP) has been successful in bone regeneration. Direct loading offers a simple means of generating a burst release and elevating the local concentration. Schoonraad et al. developed a novel MMP-cleavable peptides-based hydrogel via the modification of BMP-2 with the thiol group [111]. In this way, BMP-2 could be tethered into the hydrogel, which was composed of PEG-NB crosslinked with MMP-cleavable peptide (GCVPLSLYSGC), and which functioned well in terms of enhancing the osteogenesis of cells via the SMAD 1/5/8 pathway in the 3D microenvironment.

**Figure 6:**
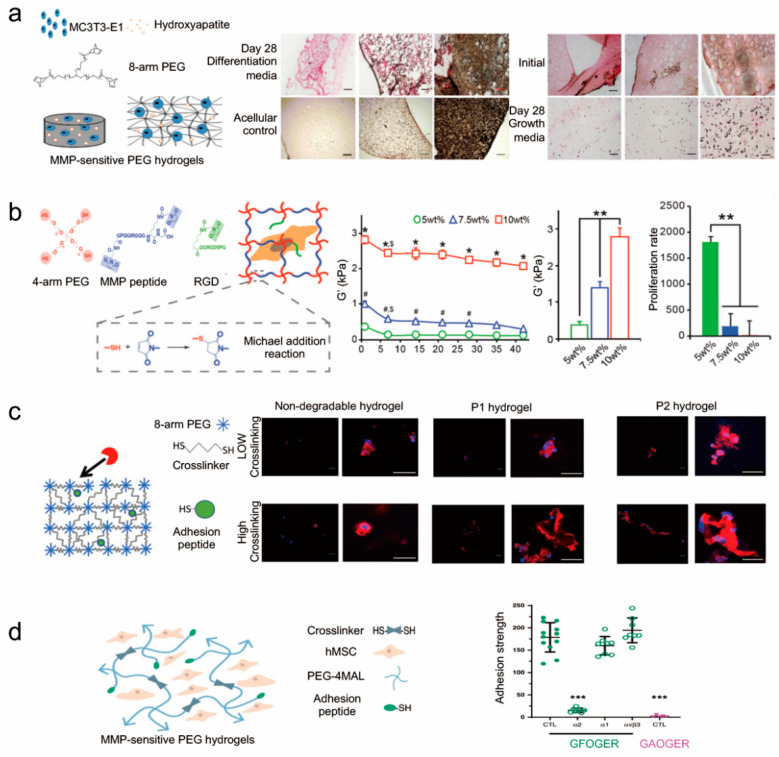
Hydrogel-based 3D in vitro models for studying cellular responses. (**a**) The nHAPs have also been embedded in MMP-cleavable peptides-based hydrogels to promote osteogenesis [113]. Reprinted with permission from Copyright © 2018 IOP Publishing Ltd. (**b**) The stiffness of MMP-cleavable peptides-based hydrogels influenced cell proliferation [105]. * *p* < 0.0001 for 10 wt% relative to 5 and 7.5 wt% at a given time point, # *p* < 0.05 for 7.5 wt% relative to 5 wt% at a given time point, $ *p* < 0.05 for a given time point relative to day 1, and ** *p* < 0.01 [105]. Reprinted with permission from Copyright © 2020 WILEY-VCH Verlag GmbH & Co. KGaA, Weinheim. (**c**) The degradation rates of MMP-cleavable peptides-based hydrogels are critical to accelerating early osteogenesis [121]. Reprinted with permission from Copyright © 2019 John Wiley and Sons. (**d**) Adhesive peptides of MMP-cleavable peptides-based hydrogels enhance the adhesion strength of cells [122]. ANOVA (*p* < 0.0001) was used to detect statistical differences followed by Sidak’s multiple comparisons test with adjustment for multiple comparisons, *** *p* < 0.0001 vs. GFOGER CTL. Reprinted with permission from Copyright © 2020 Nature Publishing Group.

In addition, some biomaterials were incorporated with MMP-degradable hydrogels to accelerate bone repair (Figure 6a). For example, hydroxyapatite nanoparticles (nHAPs) are often applied in biomaterials for bone tissue engineering due to their advanced performance in bone regeneration [112]. A nHAP-embedded MMP-degradable hydrogel was constructed by crosslinking PEG-NB with peptide crosslinker (CVPLSLYSGC) and was shown to be able to encapsulate functional live cells under UV light [113]. Including the evaluation of alkaline phosphatase (ALP) activity and cellular morphology after 28 days of cell culture, the results claimed that osteogenesis was enhanced. Thus, the biomaterial contained PEG, peptide crosslinker, RGD peptide and nHAP exhibited potential for bone regeneration.

#### 5.1.2. Promotion of Cell Spreading

The degradation of hydrogels provides space for cells to adhere and migrate [68]. Cell migration can be enhanced in the MMP-degradable hydrogel [114]. An enzymatically degradable hydrogel-crosslinked norbornene-functionalized alginate with MMP-cleavable and RGD peptides under UV was developed to culture cells [17]. The 3D synthetic environment can not only maintain cell viability for over 2 weeks, but also promote cell spreading. 

It has been demonstrated that physical cues could impact the fates of cells, including spreading and differentiation (Figure 6b). The stiffness of hydrogels was reported to promote cell differentiation [115]. Hydrogels with variable stiffness can be synthesized by changing the concentration of polymers or the density of crosslinkers [103,105]. Unfortunately, the dense networks may reduce the degradation rate and provide little space for cells to migrate [116]. Moreover, it was found that the increased stiffness would result in a decreased speed of migration due to an increased physical barrier [117]. Wei et al. designed a soft hydrogel that was crosslinked PEG-MAL with MMP-peptides. The cells encapsulated in hydrogel could proliferate to obtain enough cells that maintain the osteogenic differentiation potential with bone morphogenetic protein-2 (BMP-2) and migrate to the interface of bone defect to induce osteogenesis [103]. Furthermore, it was found that YAP (yes-associated protein) could promote osteogenesis [118]. In degradable hydrogels, YAP/TAZ signaling is not only regulated by stiffness, but is also sensitive to other parameters, such as dimensionality and degradability [119]. Meanwhile, the stiffness and roughness of hydrogels would change when the hydrogel degraded. As a result, YAP signaling pathway would be activated in MMP-cleavable peptides-based hydrogels. The soft hydrogel, which has similar properties to bone marrow stiffness, may offer an optimal strategy for bone regeneration.

Furthermore, polymers also influence cell spreading. To obtain sufficient mechanical properties, gelatin is required in a high concentration. As a result, the dense networks are too close for cells to spread. Several methods have been developed to seek a suitable structure, such as enlarging the pore sizes of hydrogels [120]. Collagen was chosen due to its complete triple helix structure and was crosslinked with peptides after modification with NB groups [83]. The collagen hydrogels possess good cell viability, spreading and proliferation with low solid and pore structures.

In addition, the degradation rates of hydrogels can impact cell behaviors (Figure 6c). Studies have revealed that cell spreading could enhance osteogenesis [49]. An MMP-sensitive PEG-NB hydrogel was developed for the spreading and osteogenesis of encapsulated human mesenchymal stem cells (hMSCs) [121]. Compared with nondegradable hydrogels, the degradable hydrogels whose degradability is mediated by cells would promote cell spreading and enhance the osteogenic capacity of hMSCs. Hydrogels composed of peptide (CVPLS↓LYSGC) are susceptible to MMP-14 and have a faster degradation compared with the hydrogels that possess the peptide (CRGRIGF↓LRTDC), resulting in faster cell migration as well as accelerated early osteogenesis. Therefore, such hydrogels exhibit promising applications for bone tissue engineering. Recently, some researchers have found that the adhesive peptide (GFOGER) not only enhances the adhesion strength, but also improves the reparative activity of BMSC (Figure 6d).

### 5.2. Hydrogels for Bone Healing

#### 5.2.1. Biodegradable Hydrogels Required for Bone Regeneration

**Figure 7:**
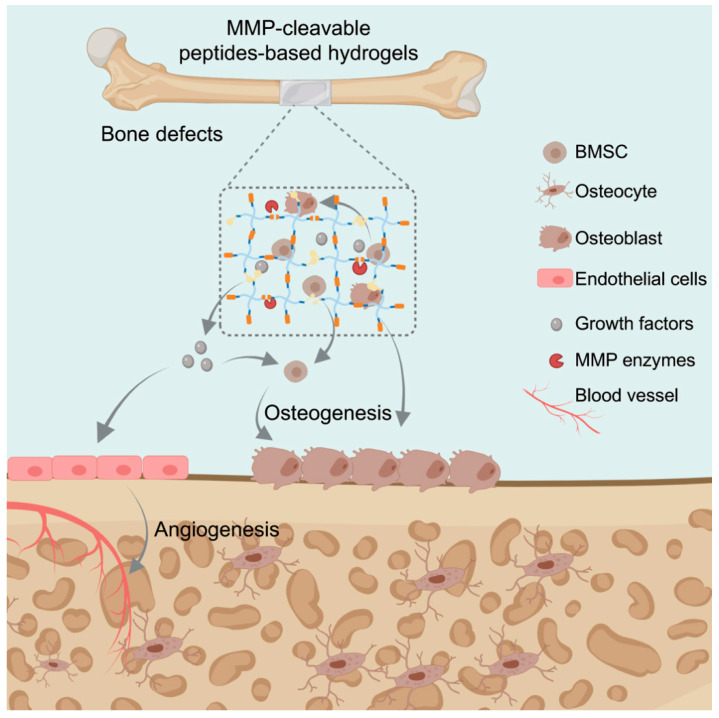
Illustration diagram of MMP-cleavable peptides-based hydrogels for bone healing.

**Figure 8:**
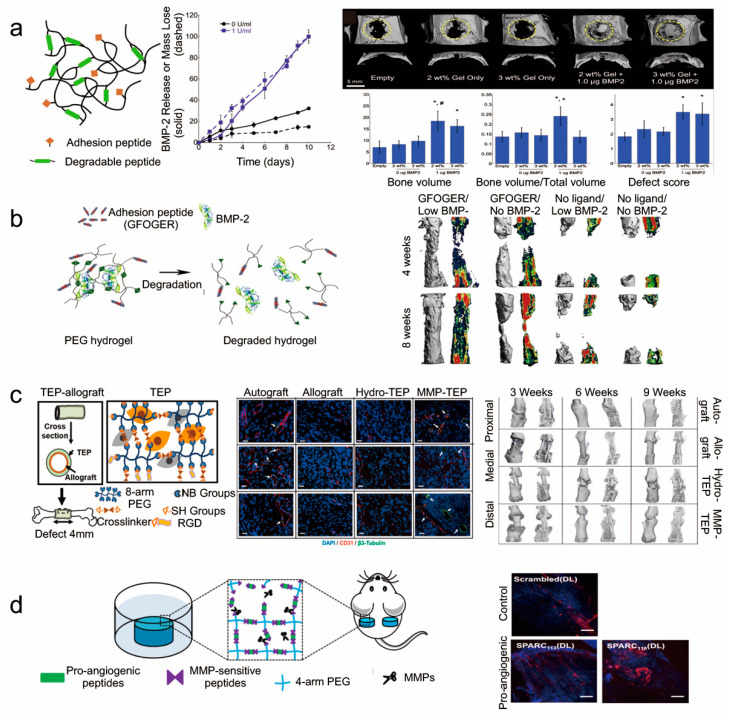
The MMP-cleavable peptides-based hydrogels for bone regeneration in vivo. (**a**) The MMP-cleavable peptides-based hydrogels were used to deliver growth factors (BMP-2) to promote bone healing [125]. Statistical significance (*p* < 0.05): (*) compared to empty defect, (#) compared to the same hydrogel formulation without BMP-2, and (+) compared to 3 wt.% hydrogels with and without BMP-2. Reprinted with permission from Copyright © 2014 Elsevier B.V. (**b**) The MMP-cleavable peptides-based hydrogels with adhesive peptide (GFOGER) enhanced bone regeneration in challenging defects [8]. Reprinted with permission from Copyright © 2014 Elsevier Ltd. (**c**) The MMP-cleavable peptides-based hydrogels were used as tissue-engineered periosteum (TEP) to coordinate bone repair via recruitment and support of host neurovasculature [30]. Reprinted with permission from Copyright © 2020 Elsevier Ltd. (**d**) The MMP-cleavable peptides-based hydrogels were explored for pro-angiogenic peptide drug delivery to increase vascularization in vivo [127]. Reprinted with permission from Copyright © 2015 Elsevier B.V.

Biodegradability was found to be necessary for the application of hydrogels in controlled therapeutic delivery as it enables noninvasive clearance and creates living space for cells [108]. After degradation, hydrogel could not only provide space for cell migration, but also release extracellular matrix (EMC) molecules that induce cell adhesion, migration and differentiation [123,124]. As a result, biodegradable hydrogels could promote bone regeneration (Figure 7). Recently, Kim et al. developed a novel type of degradable hydrogel made of chitosan and lysozyme through visible light [68]. They demonstrated that the degradation of this chitosan hydrogel was conducted by combining lysozyme, and it promoted bone formation. As it is known that MMPs play important roles in bone remodeling, several MMP-cleavable hydrogels have been developed for bone tissue engineering recent years (Figure S1A,B). Furthermore, some hydrogels have been designed to be adhesive, and it was found that they could adhere to the bone and, thus, be maintained in situ (Figure S1E). HA was found to be a component of the bone matrix and is considered as an ideal material. The novel HA-based hydrogel-containing adhesive peptide (RGD) was designed by combining MAL-HA and MMP-cleavable peptides (GCRDVPMSMRGGDRCG) via the Michael addition reaction [125]. In order to create a suitable microenvironment for bone regeneration, BMP-2 was added to the hydrogel (Figure 8a). According to the in vitro and in vivo evaluations, the hydrogel showed upregulated osteogenic gene expression and excellent bone regeneration ability.

More and more researchers have found that the RGD peptide in MMP-degradable hydrogels has vital advantages in terms of cell adhesion and spreading [125]. For instance, MMP-cleavable hydrogels grafted with RGD-adhesive peptide could improve the osteogenic capability (Figure 8b). Recently, another adhesive peptide (GFOGER) showed greater bone formation than RGD due to the intrinsic osteoinduction activity of GFOGER [8]. An α2β1 integrin-specific MMP-cleavable hydrogel was synthesized by introducing GFOGER or RGD-adhesive peptide [122]. The hMSC-loaded GFOGER hydrogel maintained hMSC activity for a long time, upregulated host angiogenic and osteogenic gene expression, and shifted the secretion profile to promote bone regeneration. The hydrogels were cast within 4-mm long polyimide tube sleeves (microlumen) and put into the 2.5 mm bone defect (Figure S1C,D). After implantation for 8 weeks, the bone formation was significantly accelerated in the hMSC and GFOGER peptide groups, as compared to the control groups. 

Angiogenesis and sensory nerve innervation were proven to be critical during bone repair [126]. According to this, a special type of cell-loaded hydrogel, formed by PEG-NB and an MMP-degradable crosslinker (GKKCGPQGIWGQCKKG) under UV, was designed as a biomimetic periosteum (TEP) for the treatment of bone defects [30]. It was found that MMP-TEP enhanced bone generation and neurovascularization during an early stage, as well as leading to faster cell recruitment and migration in vivo (Figure 8c). This could represent a promising means of partly replacing allografts for critically sized bone defects.

#### 5.2.2. Biodegradable Hydrogels as Delivery System

Hydrogels have been used as cell or growth factor vehicles in many fields [25,55]. For example, endothelial progenitor cells (EPCs) are applied to promote angiogenesis and growth factors manufacturing, in order to restore and maintain the bone microenvironment during regeneration. The hydrogel-containing adhesive peptide RGD was constructed by mixing PEG-VS and MMP-cleavable peptide solution, which carried EPCs and the growth factors vascular endothelial growth factor (VEGF) and basic fibroblast growth factor (bFGF) [59]. The growth factors were released after hydrogel degradation and promoted EPC differentiation, thus accelerating the neovascularization process.

Various peptides were applied to accelerate vascularization [127], but the applications were obviously limited due to fast clearance and poor pharmacokinetics. In order to overcome the limitations, a stimuli-responsive peptide drug delivery system was developed to deliver and release peptides on demand, such as an MMP-cleavable hydrogel composed of PEG-NB and the enzymatically responsive IPES↓LRAG sequence [128]. The functional peptides were embedded inside the sequence, which could be crosslinked with PEG-NB after the introduction of cysteine. The hydrogel could be degraded by intrinsic MMPs and peptide drugs could be released to promote endothelial cell tube formation (Figure 8d).

## 6. Conclusions and Future Outlook

MMPs take part in numerous cell activities and are identified as environmentally responsive triggers in the design of biomaterials. As the biodegradability of filling materials has been widely proven to be beneficial for bone regeneration in the literature, it offers a promising way to apply MMP-cleavable peptides-based hydrogels in bone tissue engineering. In this review, we summarized the polymers, degradable property, modified groups, reactions of hydrogels and applications of MMP-cleavable peptides-based hydrogels in bone tissue engineering.

Varieties of polymers have been identified to synthesize MMP-degradable hydrogels. In fact, different polymers have varied characteristics and advantages, as well as chemical groups and reactions. For example, due to its mild reaction conditions and fast gelatin rate, the NB group is usually adopted for 3D printing as a bio-ink via the thiol-ene photo-click chemistry reaction. Biodegradability plays a critical role in bone repair and its subsequent regeneration, and thus, MMP-cleavable hydrogels have been designed and widely used to fill bone defects and degrade appropriately. At the cellular level, the degradable hydrogels could promote cell spreading and enhance osteogenic capability. In summary, MMP-cleavable hydrogels accelerated bone formation rates via the delivery of growth factors and through their adaptive degradation rates under metabolic conditions, and thus, show great potential prospects in the regenerative field.

However, the degradation rates of peptides are diverse from each other and susceptible to different MMP subtypes. As the intercellular microenvironment is complex and dynamically changing, there may be more than one type of MMP in the regeneration site. Degradation rates might be altered inside different tissues or under different pathological conditions. As a result, it is rather difficult to propose the most accurate peptide for regenerative medicine. In addition, a great deal of polymers have been crosslinked with functional peptides to form dual-network hydrogels, whereby degradation rates could be further optimized. Last but not least, the crosslink density, concentration and molecular weight of the polymers are also critical factors that alter the degradation rates. Although there are still challenges to be honored, there is no doubt that the MMP-cleavable peptides-based hydrogels deserve further investigation and possess a rather promising future in the bone regeneration area.

## Supplementary Materials

The following are available online at https://www.mdpi.com/article/10.3390/gels7040199/s1, Figure S1: The MMP-Cleavable peptides-based hydrogels and the process of implantation.
