# Supplementary material for: Bone Regeneration Using MMP-Cleavable Peptides-Based Hydrogels
Source: Gels. 2021 Nov 5;7(4):199. doi: 10.3390/gels7040199 (PMC8628702; doi:10.3390/gels7040199)
Supplement: Supplementary file 1 [file gels-07-00199-s001.zip › gels-1430923-supplementary.pdf]

Bone Regeneration Using MMP-Cleavable Peptides-Based Hydrogels

Weikai Chen [1,2,3,†], Ziyang Zhou [1,2,4,†], Dagui Chen [1,†], Yinghua Li [1,*], Qin Zhang [1,*] and Jiacan Su [5,6,*]

[1] Institute of Translational Medicine, Shanghai University, Shanghai 200444, China; chenweikai0819@shu.edu.cn (W.C.); Zzy21724887@shu.edu.cn (Z.Z.); dagui1106@shu.edu.cn (D.C.)
[2] School of Medicine, Shanghai University, Shanghai 200444, China
[3] School of Environmental and Chemical Engineering, Shanghai University, Shanghai 200444, China
[4] School of Life Sciences, Shanghai University, Shanghai 200444, China
[5] Department of Trauma Orthopedics, Changhai Hospital, Naval Medical University, Shanghai 200433, China
[6] Shanghai Clinical Research Center for Aging and Medicine, Shanghai, 200040, China

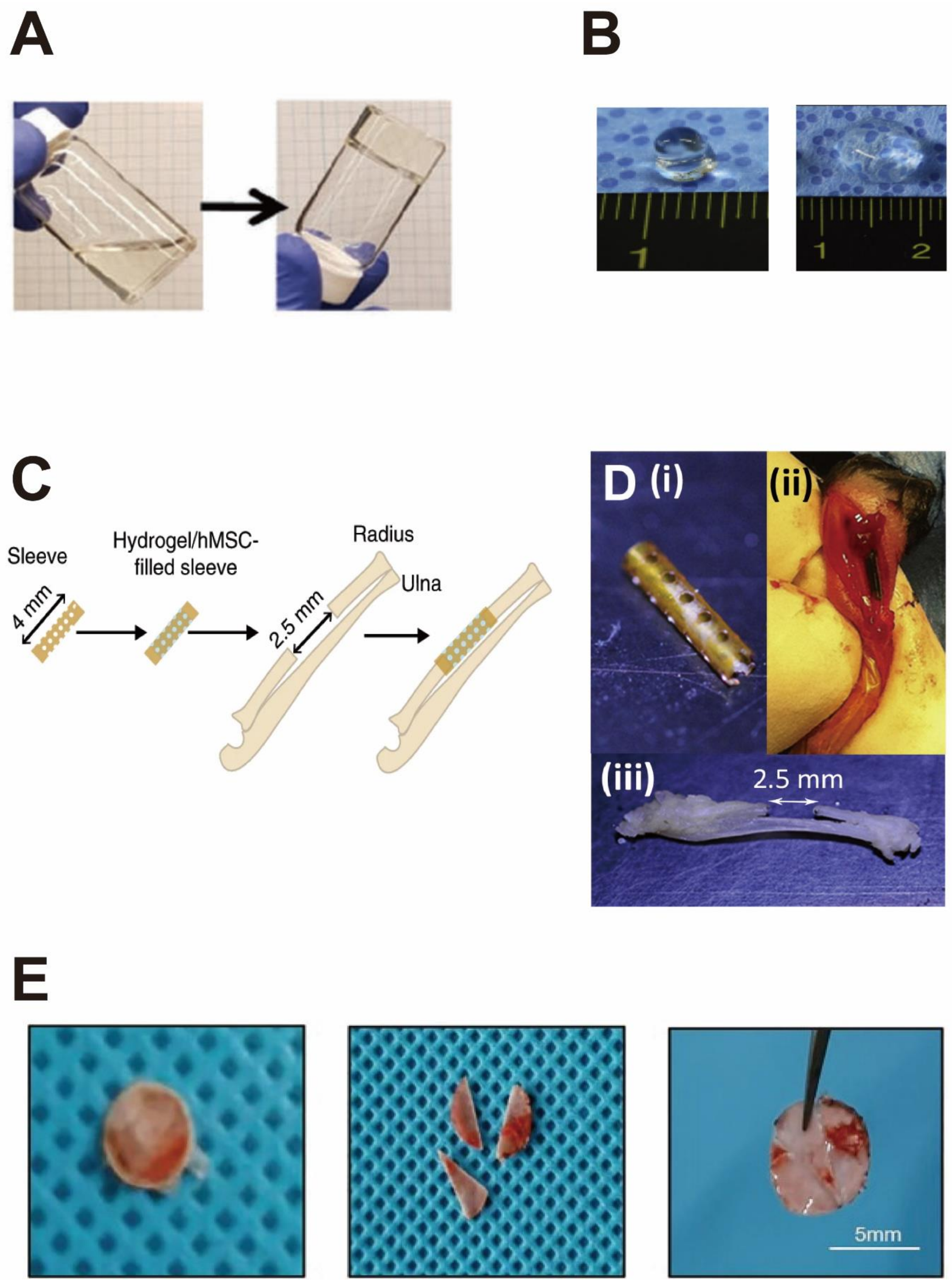

Figure S1. The MMP-Cleavable peptides-based hydrogels and the process of implantation. (A) The gelation of the MMP-Cleavable peptides-based hydrogel[1]. Reprinted with permission from Copyright © 2017 WILEY-VCH Verlag GmbH & Co. (B) The hydrogel before and after swelling[2]. Reprinted with permission from Copyright © 2013 Elsevier Ltd. (C) The illustration of hydrogels implantation[3]. Reprinted with permission from Copyright © 2020 Nature Publishing Group. (D)The hydrogels implanted into the bone defect[4]. Reprinted with permission from Copyright © 2014 Elsevier Ltd. (E) The fixed bone fragments by adhesive hydrogel[5]. Reprinted with permission from Copyright © 2021 Elsevier B.V.

References

1. Wei, Z.; Lewis, D.M.; Xu, Y.; Gerecht, S. Dual Cross-Linked Biofunctional and Self-Healing Networks to Generate User-Defined Modular Gradient Hydrogel Constructs. *Adv Healthc Mater* **2017**, *6*.
2. Song, M.; Jang, H.; Lee, J.; Kim, J.H.; Kim, S.H.; Sun, K.; Park, Y. Regeneration of chronic myocardial infarction by injectable hydrogels containing stem cell homing factor SDF-1 and angiogenic peptide Ac-SDKP. *Biomaterials* **2014**, *35*, 2436-2445.
3. Clark, A.Y.; Martin, K.E.; Garcia, J.R.; Johnson, C.T.; Theriault, H.S.; Han, W.M.; Zhou, D.W.; Botchwey, E.A.; Garcia, A.J. Integrin-specific hydrogels modulate transplanted human bone marrow-derived mesenchymal stem cell survival, engraftment, and reparative activities. *Nat Commun* **2020**, *11*, 114.
4. Shekaran, A.; Garcia, J.R.; Clark, A.Y.; Kavanaugh, T.E.; Lin, A.S.; Guldberg, R.E.; Garcia, A.J. Bone regeneration using an alpha 2 beta 1 integrin-specific hydrogel as a BMP-2 delivery vehicle. *Biomaterials* **2014**, *35*, 5453-5461.
5. Hou, F.; Jiang, W.; Zhang, Y.; Tang, J.; Li, D.; Zhao, B.; Wang, L.; Gu, Y.; Cui, W.; Chen, L. Biodegradable dual-crosslinked adhesive glue for fixation and promotion of osteogenesis. *Chemical Engineering Journal* **2022**, *427*.
